# Suprabasin-derived bioactive peptides identified by plasma peptidomics

**DOI:** 10.1038/s41598-020-79353-4

**Published:** 2021-01-13

**Authors:** Tomomi Taguchi, Yoshio Kodera, Kazuhito Oba, Tatsuya Saito, Yuzuru Nakagawa, Yusuke Kawashima, Masayoshi Shichiri

**Affiliations:** 1 Department of Endocrinology, Diabetes and Metabolism, Kitasato University School of Medicine, 1-15-1 Kitasato, Minami-ku, Sagamihara, Kanagawa 252-0374 Japan; 2 Department of Physics, Kitasato University School of Science, 1-15-1 Kitasato, Minami-ku, Sagamihara, Kanagawa 252-0373 Japan; 3 Center for Disease Proteomics, Kitasato University School of Science, 1-15-1 Kitasato, Minami-ku, Sagamihara, Kanagawa 252-0373 Japan

**Keywords:** Proteomics, Peptides, Metabolic syndrome, Inflammation

## Abstract

Identification of low-abundance, low-molecular-weight native peptides using non-tryptic plasma has long remained an unmet challenge, leaving potential bioactive/biomarker peptides undiscovered. We have succeeded in efficiently removing high-abundance plasma proteins to enrich and comprehensively identify low-molecular-weight native peptides using mass spectrometry. Native peptide sequences were chemically synthesized and subsequent functional analyses resulted in the discovery of three novel bioactive polypeptides derived from an epidermal differentiation marker protein, suprabasin. SBSN_HUMAN[279–295] potently suppressed food/water intake and induced locomotor activity when injected intraperitoneally, while SBSN_HUMAN[225–237] and SBSN_HUMAN[243–259] stimulated the expression of proinflammatory cytokines via activation of NF-κB signaling in vascular cells. SBSN_HUMAN[225–237] and SBSN_HUMAN[279–295] immunoreactivities were present in almost all human organs analyzed, while immunoreactive SBSN_HUMAN[243–259] was abundant in the liver and pancreas. Human macrophages expressed the three suprabasin-derived peptides. This study illustrates a new approach for discovering unknown bioactive peptides in plasma via the generation of peptide libraries using a novel peptidomic strategy.

## Introduction

Human plasma is the most commonly sampled diagnostic biospecimen and is a potentially informative resource for characterizing proteomes^1^. Low-molecular-weight peptides that circulate in plasma as hormones, cytokines and growth factors, constitute an essential part of the homeostatic regulatory mechanism of many physiological processes. Despite recent successes in tryptic plasma-based proteome analyses^2,3^, an important unmet challenge is to comprehensively identify plasma native peptide sequences that have undergone endogenous proteolytic processing^4^. Polypeptide sequences stored in protein databases consist almost exclusively of trypsin-digested sequence information of proteins derived from tissues/organs, secreted products and some biological fluids^1^. The enormous concentration range of currently identified plasma proteins/peptides roughly spans 12 orders of magnitude from highly expressed proteins to low-abundance bioactive/biomarker peptides^4,5^. This makes direct in-depth plasma peptidomics profiling one of the most challenging tasks in analytical biochemistry^6,7^ and represents a major limiting factor for identifying bioactive/biomarker peptides and their receptors. Our previous approach to identify novel bioactive peptides used full-length cDNA library information and the synthesis of putative native peptide sequences estimated from the proteolytic processing of secretory proteins. We then explored the biological activities of these peptides and confirmed their immunoreactive presence in human peripheral circulation^8,9^. This in silico methodology enabled the discovery of potent bioactive peptides possessing physicochemical characteristics that prevent their identification by conventional methods: however, to confirm their discovery, exact native amino acid sequences in the peripheral circulation need to be demonstrated^10^.

We attempted to improve our high-yield plasma extraction technique^11^ to allow comprehensive identification of undiscovered native peptides using mass spectrometry without enzyme-digestion of plasma proteins. Our original method, which we named “differential solubilization”, efficiently depleted plasma of highly abundant proteins with minimal loss of low-molecular-weight peptides. This efficiently enriched native peptides that were both unbound and bound to carrier proteins, but not to the level required to detect important low-molecular-weight bioactive peptides by mass spectrometry. Here we describe optimization of the protocol to further remove residual plasma proteins from extracts enriched by the above method and to improve the efficiency of peptide enrichment to such an extent as to enable large-scale identification of endogenously processed low-molecular-weight peptides in human peripheral circulation^12^.

Suprabasin, originally identified in mouse and human differentiating keratinocytes^13^, is an epidermal differentiation marker detected in the suprabasal layers of the epithelia of the tongue, stomach and epidermis, indicating a potential role in epidermal differentiation^14^. Suprabasin is highly expressed in several human solid malignancy^15,16^, bone marrow cells^17^ and tumor endothelium^18^, contributes to cancer progression, invasiveness and metastasis^15,16,19,20^ and is hypothesized to be a proto-oncogene candidate of still unknown function^19^. The power of our peptidomic approach for identifying bioactive peptides is illustrated by the discovery of suprabasin-derived bioactive peptides in human plasma.

## Results

### Peptidomic strategy for the analysis of non-tryptic human plasma

We used 2–200 μL of pooled human plasma from four healthy individuals to deplete high-abundant proteins and to further remove residual high-molecular weight proteins using our modified “differential solubilization” methodology^11,12^. We confirmed using tricine SDS–polyacrylamide gel electrophoresis that this procedure maximally enriched for low-molecular weight native peptides that had been both bound and unbound to plasma carrier proteins as described^11,12^. Enriched eluates, with or without the reductive alkylation procedure, were either analyzed directly without prefractionation by liquid chromatography tandem-mass spectrometry (LC–MS/MS) or subjected to reversed-phase high performance liquid chromatography (RP-HPLC) for prefractionation into 8 or 13 fractions by a ‘cyclic sample pooling technique’ prior to LC–MS/MS^21^. To minimize hydrophobic peptide loss, we used siliconized tubes in all procedures and LC–MS/MS compatible surfactants for repetitive enrichment and solvent exchange during lyophilization and redissolution^22^. Subsequent LC–MS/MS analyses of both unseparated extracts and those prefractionated yielded high resolution/high sensitivity data, which were subjected to a PEAKS database search based on a de novo sequencing-based database search^10^. The above process facilitated the direct detection of plasma native peptides at concentrations as low as picomolar range. This resulted in the identification of more than 7959 distinct native peptide sequences with peptide identification false discovery rates (FDR) of 0% after excluding peptides derived from the keratin protein family. The peptidomics data identified by mass spectrometry have been deposited into the ProteomeXchange Consortium via the PRIDE^23^ partner repository with the dataset identifier PXD003533, which contains other peptides identified by a PEAKS PTM search in addition to the 7959 peptides identified.

### Synthetic polypeptides of the identified novel sequences

We sought to discover novel bioactive peptides using synthetic polypeptides designed from the identified sequences. We selected native peptide sequences that were less than 39 amino acid residues in length, identified with an FDR of 0%, uniquely assigned to a single secretory protein, and without any amino acid substitutions/modifications. Of the 104 peptides we initially synthesized, nine showed insufficient solubility and/or low purity by LC–MS/MS analysis. Thus, the remaining 95 peptides (Supplementary Table S1) were tested for their ability to induce cellular responses in cultured mammalian cells or to modulate spontaneous animal behaviors as described below.

### Mitogenic and anti-apoptotic effects of SBSN_HUMAN[225–237] and SBSN_HUMAN[243–259]

**Figure 1:**
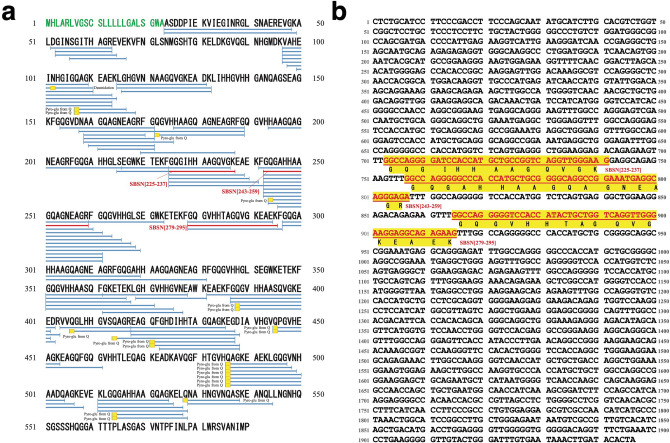
Peptidomic identification of suprabasin-derived native peptides in healthy human plasma. (**a**) Suprabasin and its cleaved peptide products comprehensively identified by LC–MS/MS. Red horizontal lines indicate the three detected sequences that we have shown to be potent bioactive peptides. Blue horizontal lines indicate cleaved products of prosuprabasin protein in human plasma identified with an FDR of 0% by our peptidomic analysis. The small yellow boxes denote amino acids that underwent modification. The predicted signal peptides of the preproproteins are shown in green letters. (**b**) The nucleotide sequence encoding suprabasin and the deduced amino acids of the three mature peptides. The nucleotide sequences corresponding to the three mature suprabasin-derived peptides are underlined in red and highlighted in yellow.

**Figure 2:**
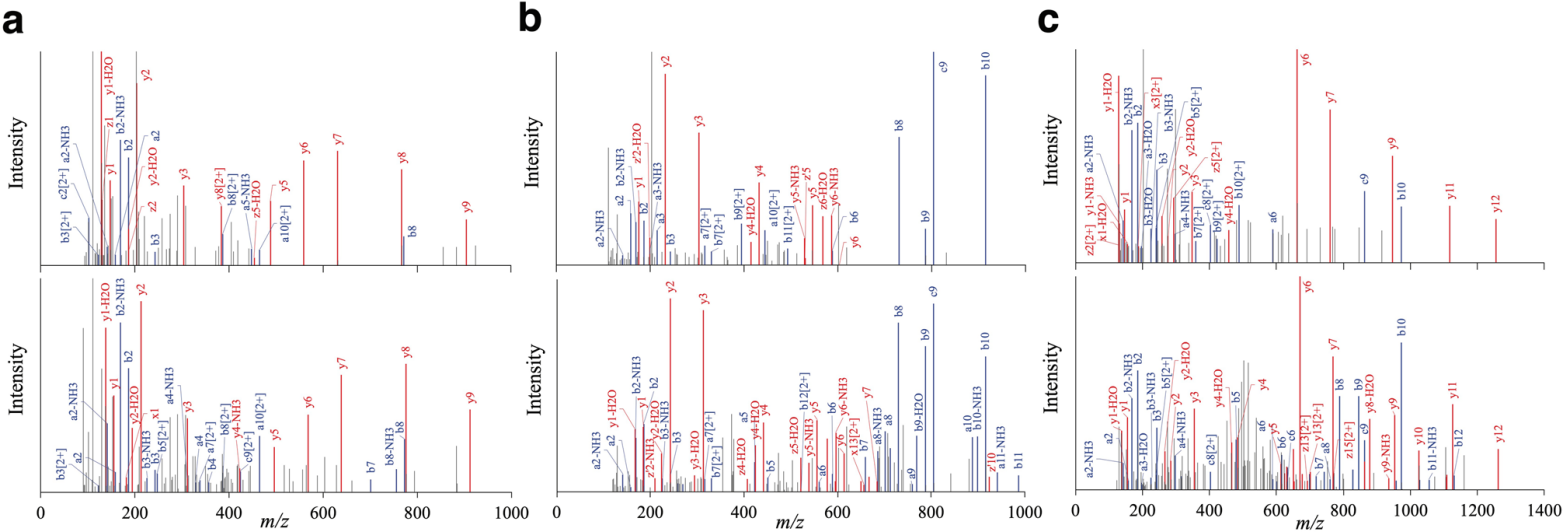
Annotated MS/MS fragmentation spectra for suprabasin-derived peptides filtered by peptidomic analysis and comparisons with those of corresponding stable isotope-labeled synthetic peptides. Human plasma extracted using the improved differential solubilization method was prefractionated and subjected to LC–MS/MS analysis. MS/MS spectra with sequence assignments of fragment ions corresponding to (**a**) SBSN_HUMAN[225–237] “GQGIHHAAGQVGK” with a *m/z* 420.5583 (*z* = 3), (**b**) SBSN_HUMAN[243–259] “GQGAHHAAGQAGNEAGR” with a *m/z* 397.9386 (*z* = 4) and (**c**) SBSN_HUMAN[279–295] “GQGVHHTAGQVGKEAEK” with a *m/z* 578.2957 (*z* = 3) (upper panels) are compared with those of respective stable isotope-labeled synthetic peptides (lower panels) to confirm the putative identification. MS/MS spectra were deconvoluted into singly charged ions from the observed spectra and peaks were assigned theoretical *m/z* values for fragment ions. The annotations of the identified matched amino terminus-containing ions are shown in blue and the carboxyl terminus-containing ions in red. The *m/z* differences between theoretical and observed values for most assigned peaks were less than 0.01 Da.

**Figure 3:**
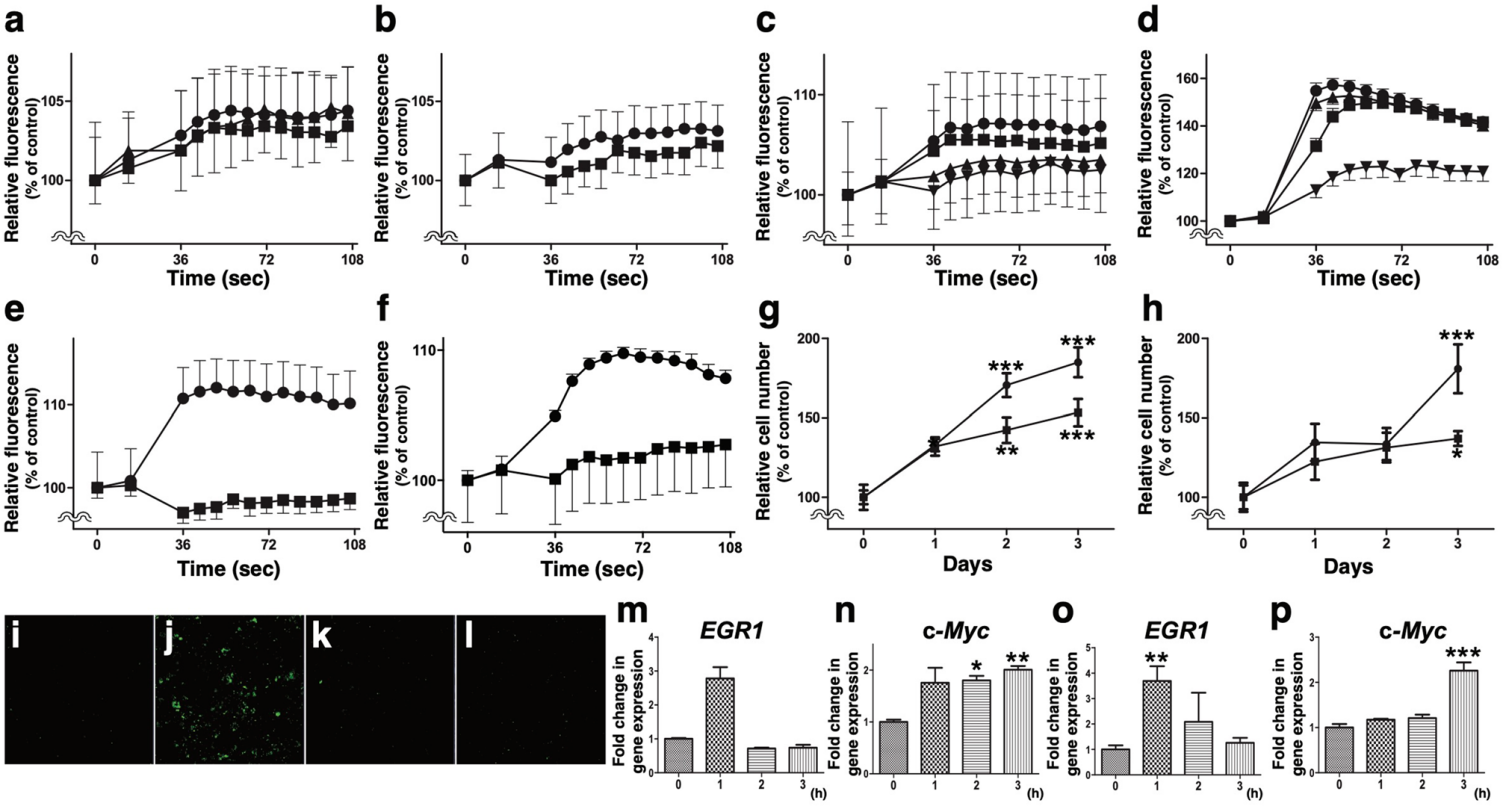
Intracellular responses of vascular smooth muscle cells to SBSN_HUMAN[225–237] and SBSN_HUMAN[243–259]. (**a**–**f**) Effect of SBSN_HUMAN[225–237] and SBSN_HUMAN[243–259] on [Ca^2+^]_i_. A10 cells (**a**,**c**) and HAoSMCs (**b**,**d**) were stimulated with either SBSN_HUMAN[225–237] (**a**,**b**) or SBSN_HUMAN[243–259] (**c**,**d**) (closed circle: 10^–6^ M; closed square: 10^–7^ M, closed triangle: 10^–8^ M, closed inverted triangle: 10^–9^ M) and Fluo-4/AM fluorescence intensities were monitored. A10 cells (**e**) and HAoSMCs (**f**), pretreated without (closed circle) or with 10^–5^ M nicardipine (closed square), were stimulated with SBSN_HUMAN[243–259]. (**g**,**h**). Data represent the mean ± S.E.M of octuple assays. (**g**,**h**) Effect of SBSN_HUMAN[225–237] (**g**) or SBSN_HUMAN[243–259] (**h**) on A10 cell proliferation (closed circle: 10^–6^ M, closed square:10^–7^ M). *p < 0.05, **p < 0.01, ***p < 0.01 compared with day 0. Data represent the mean ± S.E.M of octuple assays and are expressed relative to control cells. (**i**–**l**) Inhibitory effect of SBSN_HUMAN[225–237] or SBSN_HUMAN[243–259] on serum-deprivation-induced apoptosis in HAoSMCs. HAoSMCs were incubated in culture medium supplemented with 10% serum (**i**) or deprived of serum for 9 h without (**j**) or with the addition of either SBSN_HUMAN[225–237] (10^–6^ M) (**k**) or SBSN_HUMAN[243–259] (10^–6^ M) (**l**). Cells were fixed and enzymatic labeling of DNA strand breaks was visualized by the TUNEL assay (green). (**m**–**p**) Time course of immediate early response gene upregulation. HAoSMCs were deprived of serum for 16 h and *EGR1* and c-*Myc* mRNA levels were quantified after addition of SBSN_HUMAN[225–237] (10^–7^ M) (**m**,**n**) or SBSN_HUMAN[243–259] (10^–7^ M) (**o**,**p**) for the indicated times. Data represent the mean ± S.E.M of the percentage of mRNA copies relative to untreated cells (0 h) from six assays. *p < 0.05, **p < 0.01, ***p < 0.01 compared with 0 h.

We first tested the abilities of the 95 synthetic peptides to elicit biological responses in cultured mammalian cells. We found that a 13 amino acid residue peptide, GQGIHHAAGQVGK {SBSN_HUMAN[225–237] or suprabasin(225–237), monoisotopic mass 1259.6603} (Figs. 1a,b, 2a) and a 17 amino acid residue peptide, GQGAHHAAGQAGNEAGR {SBSN_HUMAN[243–259] or suprabasin(243–259), monoisotopic mass 1588.7323} (Figs. 1a,b, 2b), both derived from an epidermal differentiation marker protein, suprabasin^14^, induced sustained increases in intracellular free Ca^2+^ levels ([Ca^2+^]_i_) in rat vascular smooth muscle (A10) cells (Fig. 3a,c) and human aortic smooth muscle cells (HAoSMCs) (Fig. 3b,d). The effect of SBSN_HUMAN[243–259] on [Ca^2+^]_i_ increase in A10 cells and HAoSMCs was concentration-dependent and more potent (Fig. 3c,d) than that of SBSN_HUMAN[225–237] (Fig. 3a,b), and was blocked by pretreatment with a dihydropyridine-sensitive calcium channel antagonist, nicardipine (Fig. 3e,f). Both SBSN_HUMAN[225–237] and SBSN_HUMAN[243–259] stimulated the proliferation of quiescent A10 cells (Fig. 3g,h). Treatment of HAoSMCs with SBSN_HUMAN[225–237] or SBSN_HUMAN[243–259] decreased the number of apoptotic cells induced by serum deprivation (Fig. 3i–l), indicating their anti-apoptotic effect. SBSN_HUMAN[225–237] and SBSN_HUMAN[243–259] induced expressions of the immediate early genes, *EGR1* and c-*Myc*, in HAoSMCs (Fig. 3m–p). Our peptidomic analysis identified 119 suprabasin-derived cleaved peptides with an FDR of 1% and 96 peptides with an FDR of 0% (Fig. 1a). Among the 95 initially synthesized peptides were three other suprabasin-derived peptides, SBSN_HUMAN[423–438], SBSN_HUMAN[511–524] and SBSN_HUMAN[511–525], but none of them showed any similar biological activities.

**Figure 4:**
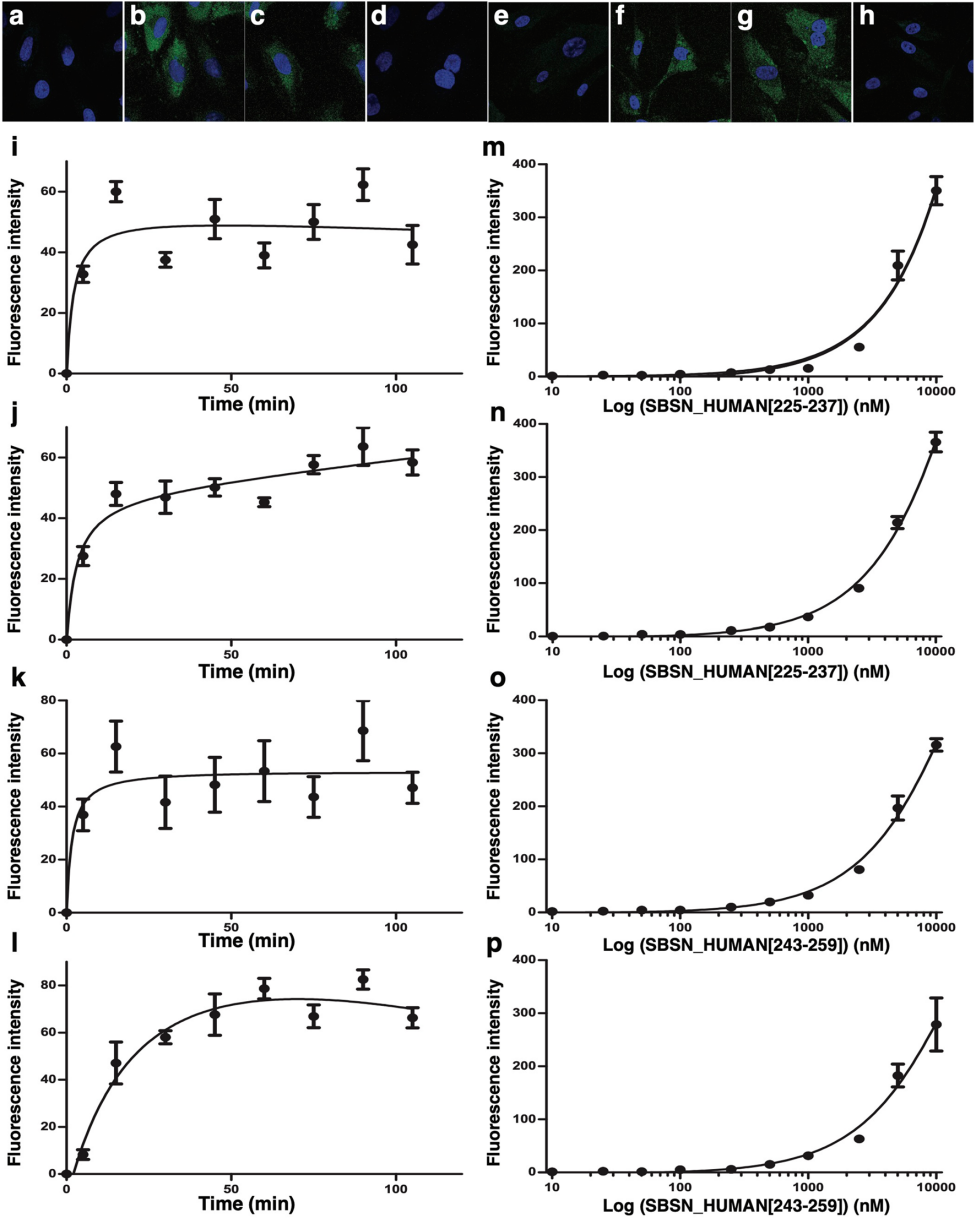
Binding of suprabasin-derived peptides to HAoSMCs. (**a**–**h**) Confocal laser-scanning microscopy images of fluorescent suprabasin-derived peptides bound to cultured A10 cells and HAoSMCs. Growing A10 cells (**a**–**d**) and HAoSMCs (**e**–**h**) were deprived of serum for 16 h and overlayed without (**a**,**e**) or with 10^–6^ M FAM-SBSN_HUMAN[225–237] (**b**,**f**), FAM-SBSN_HUMAN[243–259] (**c**,**g**), or FAM-SBSN_HUMAN[279–295] (**d**,**h**) for 30 min. Cells were washed, fixed, the nuclei counterstained with DAPI (blue) and the cell surface-bound green fluorescence visualized. (**i**–**l**) Time course of FAM-SBSN_HUMAN[225–237] and FAM-SBSN_HUMAN[243–259] bound to the cell surface. FAM-SBSN_HUMAN[225–237] (**i**,**j**) or FAM-SBSN_HUMAN[243–259] (10^–6^ M) (**k**,**l**) was overlayed on confluent cultures of A10 cells (**i**,**k**) or HAoSMCs (**j**,**l**) in 96-well plates for the indicated times and, after extensive washing, cell-bound immunofluorescence was quantified. (**m**–**p**) Concentration response relationships for FAM-SBSN_HUMAN[225–237] and FAM-SBSN_HUMAN[243–259] bound to the cell surface. Confluent cultures of A10 cells (**m**,**o**) or HAoSMCs (**n**,**p**) were treated with the indicated concentrations of FAM-SBSN_HUMAN[225–237] **(m**,**n**) or FAM-SBSN_HUMAN[243–259] (**o**,**p**) for 60 min and cell-bound immunofluorescence was quantified.

To determine whether SBSN_HUMAN[225–237] or SBSN_HUMAN[243–259] bound to the surface of intact cells, we incubated A10 cells and HAoSMCs with the peptides labeled with 5-carboxyfluorescein at the N-terminus (FAM-suprabasin peptides). Confocal immunofluorescence microscopy gave no signals in the absence of a fluorescence-labeled peptide (Fig. 4a,e), but detected the presence of specific signals on the surface of A10 cells and HAoSMCs onto which FAM-SBSN_HUMAN[225–237] (Fig. 4b,f) or FAM-SBSN_HUMAN[243–259] (Fig. 4c,g) were overlaid to bind to their cell surface. FAM-SBSN_HUMAN[279–295] did not bind to these cells (Fig. 4d,h). Bindings of FAM-SBSN_HUMAN[225–237] and FAM-SBSN_HUMAN[243–259] to the surface of confluent A10 cells and HAoSMCs was detected within 15 min and reached a plateau after 50 min (Fig. 4i–l). Both FAM-SBSN_HUMAN[225–237] and FAM-SBSN_HUMAN[243–259] bound to A10 cells and HAoSMCs in a concentration-dependent fashion (Fig. 4m–p), revealing abundant binding sites in these cells.

### SBSN_HUMAN[225–237] and SBSN_HUMAN[243–259] stimulate NF-κB-mediated cytokine production

**Figure 5:**
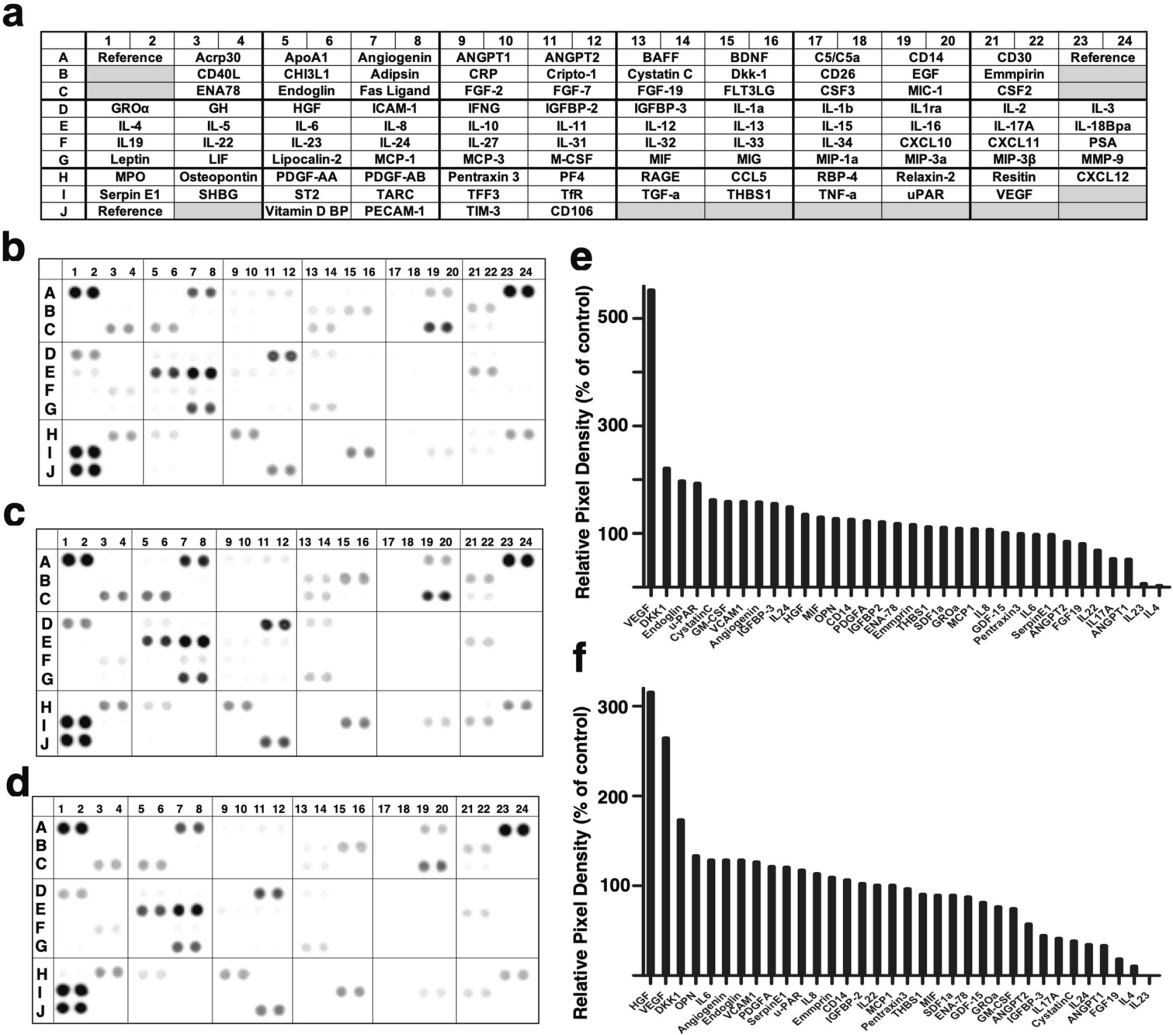
Human cytokine array screening with cultured conditioned media of HAoSMCs after stimulation with SBSN_HUMAN[225–237] or SBSN_HUMAN[243–259]. (**a**) Reference key for cytokine array, adapted from the manufacturer’s information. (**b**–**d**) Complete array images after probing with conditioned media. HAoSMCs were deprived of serum for 16 h, and incubated without (**b**) or with 10–7 M SBSN_HUMAN[225–237] (**c**) or 10–7 M SBSN_HUMAN[243–259] (**d**) for 24 h. (**e**,**f**) Immunoreactivities of respective cytokines released from HAoSMCs stimulated with either SBSN_HUMAN[225–237] (**e**) or SBSN_HUMAN[243–259] (**f**) were quantified and 2-spot mean values relative to untreated experiments are shown.

**Figure 6:**
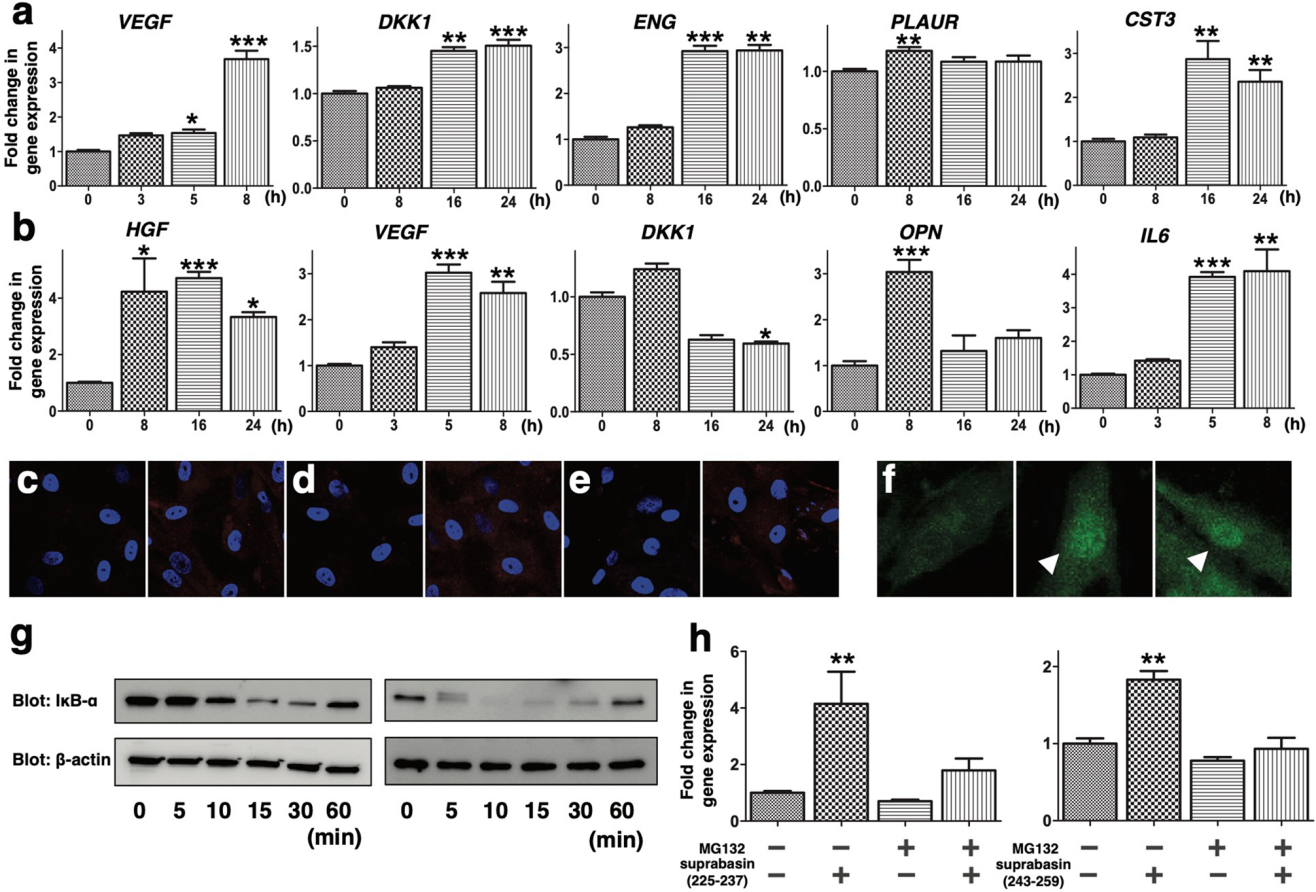
Induction of cytokine expression and activation of NF-κB by SBSN_HUMAN[225–237] and SBSN_HUMAN[243–259]. (**a**,**b**) Growing HAoSMCs were deprived of serum for 16 h and then replaced with serum-free medium containing 10^–7^ M SBSN_HUMAN[225–237] (**a**) or 10^–7^ M SBSN_HUMAN[243–259] (**b**) for the indicated times. *VEGF*, *DKK1*, *ENG*, *PLAUR*, *CST3*, *HGF*, *OPN* and *IL6* mRNA levels were quantified using real-time RT-PCR. The relative mRNA levels are shown as fold changes (mean ± S.E.M) (n = 6). *p < 0.05, **p < 0.01, ***p < 0.01 compared with 0 h. (**c**–**e**) HAoSMCs were serum-starved for 16 h and then incubated for 24 h before (left panels) and after (right panels) the addition of 10^–7^ M SBSN_HUMAN[225–237] (**c**,**d**) or 10^–7^ M SBSN_HUMAN[243–259] (**e**) and immunofluorescence staining was performed using antibodies against VEGF (**c**), HGF (**d**) and IL6 (**e**). The red signals correspond to cytokine expression. The nuclei were counterstained with DAPI (blue). (**f**) HAoSMCs were incubated without (left panel), with 10^–7^ M SBSN_HUMAN[225–237] (middle panel), or with 10^–7^ M SBSN_HUMAN[243–259] (right panel) for 2 h, and immunofluorescence staining was performed using an NF-κB p65 subunit antibody to detect its nuclear translocation (indicated with white arrowheads). (**g**) HAoSMCs were stimulated with either 10^–7^ M SBSN_HUMAN[225–237] (left panel) or 10^–7^ M SBSN_HUMAN[243–259] (right panel) for the indicated times, and subjected to western blot analysis using anti-IκB-α and anti-β-actin antibodies to assess the time-course of IκB-α degradation. The panels show the cropped blots and the full-length blots are presented in Supplementary Information. (**h**) HAoSMCs pretreated with or without MG132 (100 nM) for 30 min were incubated with SBSN_HUMAN[225–237] and SBSN_HUMAN[243–259] for 8 h and *VEGF* mRNA levels were quantified. **p < 0.01 compared with vehicle. The relative mRNA levels are shown as fold changes (mean ± S.E.M) (n = 6).

To investigate whether SBSN_HUMAN[225–237] and SBSN_HUMAN[243–259] stimulate the release of bioactive substances from vascular smooth muscle cells, we incubated HAoSMCs with or without 10^–7^ M SBSN_HUMAN[225–237] or SBSN_HUMAN[243–259]. The conditioned media were then collected and applied to protein arrays of 105 human cytokines and chemokines (Fig. 5a). Representative blots generated by this analysis are shown in Fig. 5b–d and the relative signal intensities of a variety of cytokines induced by SBSN_HUMAN[225–237] or SBSN_HUMAN[243–259] versus vehicle-treated control experiments were quantified. Addition of SBSN_HUMAN[225–237] increased the secretion of vascular endothelial growth factor (VEGF), dickkopf-related protein 1 (DKK1), endoglin (ENG), urokinase plasminogen activator receptor (uPAR), cystatin C, macrophage colony-stimulating factor 1 (GM-CSF) and vascular cell adhesion protein 1 (VCAM1), while SBSN_HUMAN[243–259] stimulated the release of hepatocyte growth factor (HGF), VEGF, DKK1, osteopontin (OPN) and interleukin (IL) 6 (Fig. 5e,f). To confirm that HAoSMCs constitutively secrete these bioactive proteins, we extracted culture supernatant of serum-starved HAoSMCs and, after enzyme-digestion and reductive alkylation, the extracted eluates were analyzed by LC–MS/MS. We identified 4121 digested peptides derived from 884 proteins with peptide identification FDR of 1%. DKK1, ENG, HGF, cystatin C, IL6, GM-CSF and VCAM1 protein sequences were detected with sequence coverage ratios of 4.9%, 7.8%, 1.1%, 30.8%, 12.3%, 5.4% and 5.0%, and peptide spectrum match scores of 1, 4, 1, 5, 3, 3 and 4, respectively (Supplementary Table S2). We next tested whether SBSN_HUMAN[225–237] or SBSN_HUMAN[243–259] can upregulate the expression of these cytokine genes in HAoSMCs. We performed quantitative RT-PCR for *VEGF*, *DKK1*, *ENG*, uPAR *(PLAUR)*, cystatin C *(CST3)*, *HGF*, *OPN*, and *IL6* in HAoSMCs stimulated with or without SBSN_HUMAN[225–237] or SBSN_HUMAN[243–259]. SBSN_HUMAN[225–237] and SBSN_HUMAN[243–259] significantly upregulated the mRNA levels of most of these genes (Fig. 6a,b). To assess whether SBSN_HUMAN[225–237]- and SBSN_HUMAN[243–259]-stimulated increases in *VEGF, HGF, IL6* mRNA levels are accompanied by enhanced levels of their respective proteins, we performed immunofluorescence staining of HAoSMCs. Pretreatment with SBSN_HUMAN[225–237] or SBSN_HUMAN[243–259] resulted in marked increases in VEGF, HGF, and IL6 protein levels compared with untreated cells (Fig. 6c–e). These results indicate that SBSN_HUMAN[225–237] and SBSN_HUMAN[243–259] are endogenous inducers of a variety of cytokines in vascular smooth muscle cells.

Most of the above cytokines markedly upregulated by SBSN_HUMAN[225–237] and SBSN_HUMAN[243–259] are either induced via the NF-κB pathway and/or possess κB-binding sites in their 5′-untranslated regions; therefore, we studied whether SBSN_HUMAN[225–237] and SBSN_HUMAN[243–259] stimulate NF-κB activity in A10 cells and HAoSMCs. HAoSMCs were treated with SBSN_HUMAN[225–237] or SBSN_HUMAN[243–259] for various lengths of time and then immunostained for the p65 subunit of NF-κB. After incubation for 30–120 min, both SBSN_HUMAN[225–237] and SBSN_HUMAN[243–259] clearly increased NF-κB immunoreactivity within the nuclei of HAoSMCs, indicating its nuclear translocation (Fig. 6f). Both SBSN_HUMAN[225–237] and SBSN_HUMAN[243–259] caused a rapid (within 5–30 min) and transient degradation of IκB-α, which then returned to baseline levels within 1 h (Fig. 6g). To determine the involvement of NF-κB signaling in SBSN_HUMAN[225–237]- and SBSN_HUMAN[243–259]-mediated VEGF expression, we pretreated HAoSMCs with a proteasome inhibitor, MG132, and stimulated the cells for 8 h with SBSN_HUMAN[225–237] or SBSN_HUMAN[243–259]. Inhibition of NF-κB by MG132 blocked the induction of VEGF expression by SBSN_HUMAN[225–237] and SBSN_HUMAN[243–259] (Fig. 6h).

### Anorexigenic and anti-dipsogenic effects of SBSN_HUMAN[279–295]

**Figure 7:**
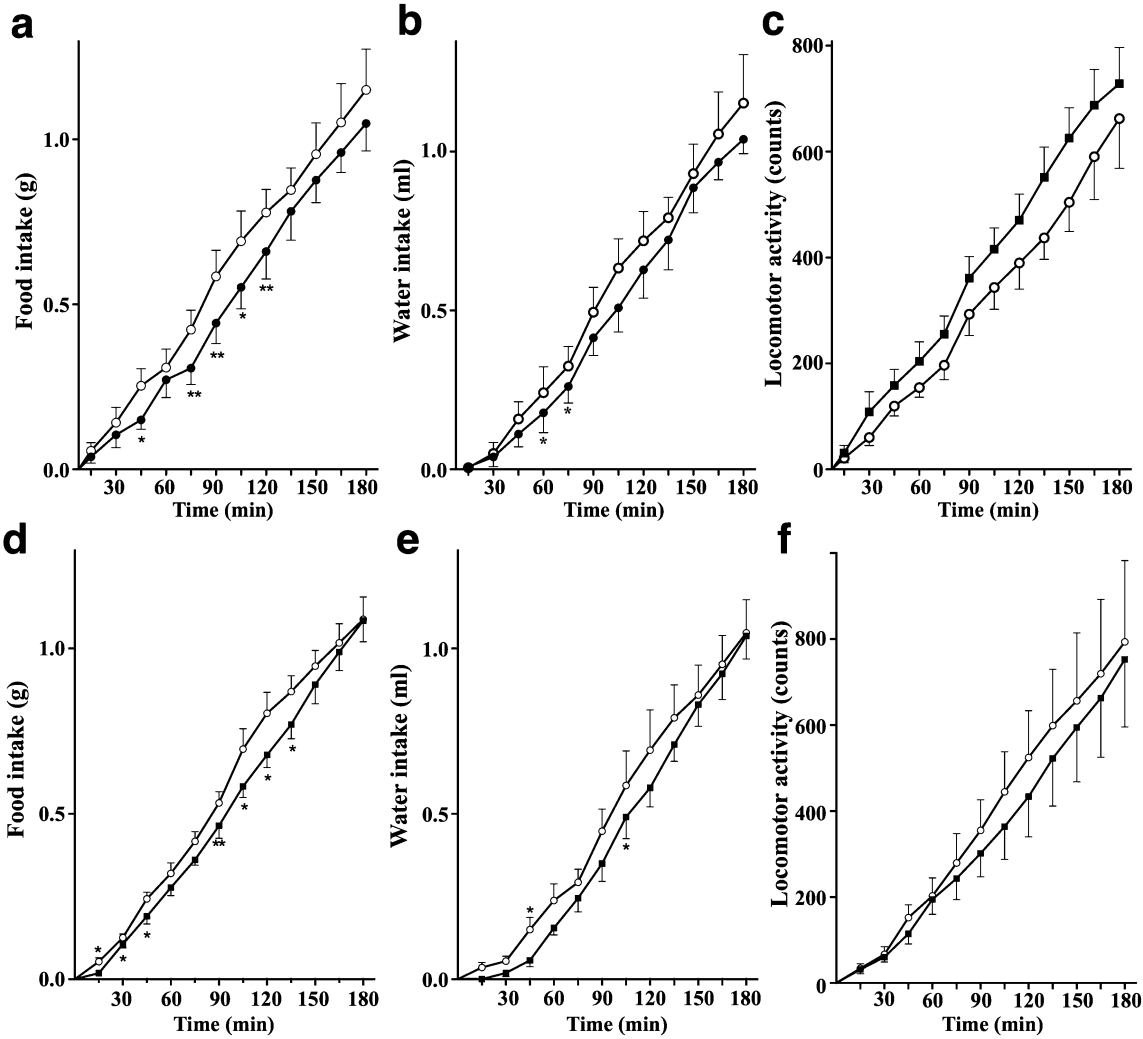
Effects of SBSN_HUMAN[279–295] on feeding, drinking and locomotor behaviors in mice. (**a**–**f**) Synthetic SBSN_HUMAN[279–295] was intraperitoneally injected to ad libitum watered and fed mice approximately 30 min before the onset of the dark phase, and cumulative food intake (**a**,**d**), water intake (**b**,**e**) and spontaneous locomotor activities (**c**,**f**) were monitored throughout the entire dark phase of the diurnal cycle. Cumulative food and water intake are expressed in grams and mL, respectively, and physical activity is represented by infrared beam interruption counts for non-treated mice (open circles), and for mice treated with 10 pmol/mouse (**a**–**c**) or with 100 pmol/mouse (**d**–**f**) SBSN_HUMAN[279–295] (closed circle) during the initial 180 min period. *p < 0.05, **p < 0.01 compared with control animals. Data are expressed as the mean ± S.E.M. (*n* = 6–7 mice per group).

We next studied whether any of the 95 synthetic peptides could regulate appetite, thirst, or spontaneous behaviors more potently than already known anorexigenic/orexigenic peptides. Mice were intraperitoneally injected with 0, 10 or 100 pmol synthetic peptide prior to the start of a dark phase and eating, drinking, and locomotor activities were continuously monitored throughout the dark phase^24,25^. We found that another 17 amino acid residue peptide derived from suprabasin, GQGVHHTAGQVGKEAEK {SBSN_HUMAN[279–295] or suprabasin(279–295), monoisotopic mass 1732.8725} (Figs. 1a,b, 2c), suppressed food and water intake and induced spontaneous locomotor activity at both 10 pmol/mouse and 100 pmol/mouse (Fig. 7, ANOVA, *p* < 0.0001). An inhibitory effect of SBSN_HUMAN[279–295] on food intake was significant as early as 15–45 min following injection, and lasted for at least 120 min. These results demonstrated SBSN_HUMAN[279–295] to be a potent, endogenous, peripherally acting anorexigenic peptide in human plasma.

### Presence of suprabasin-derived peptides in human plasma, tissues and cells

To determine accurate plasma levels of the three suprabasin-derived peptides, all of which have high hydrophilicity, we synthesized respective stable isotope labelled peptides (Fig. 2). We then spiked human plasma samples with serial dilution of the peptides prior to extraction. LC–MS/MS analyses were performed to generate the extracted ion chromatogram (XIC) intensities^26^. The plasma concentrations of SBSN_HUMAN[225–237], SBSN_HUMAN[243–259] and SBSN_HUMAN[279–295] extrapolated from the XICs generated by the respective endogenous peptides and the reference peptides revealed 0.3, 0.3 and 1.5 nM, respectively.

**Figure 8:**
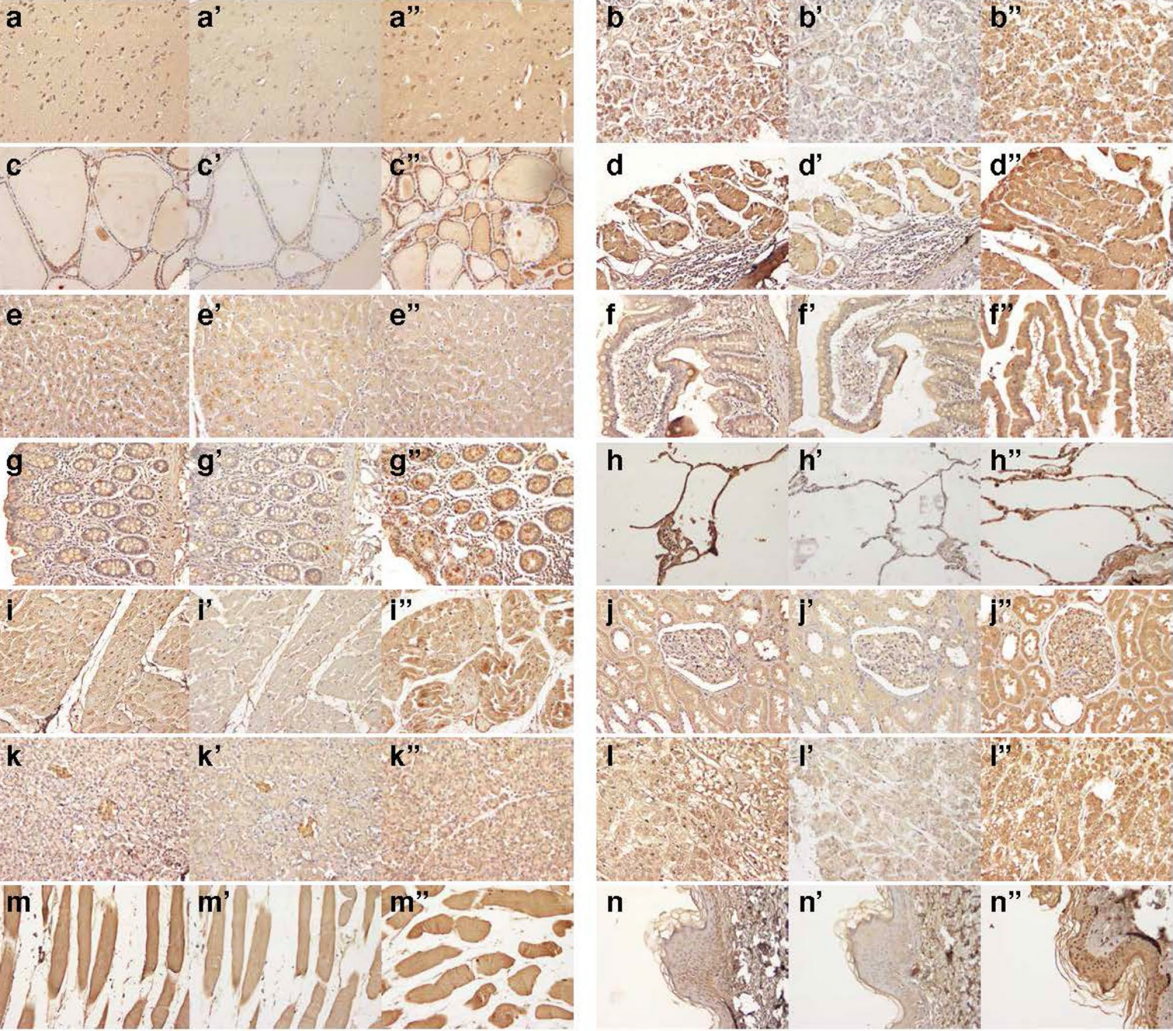
Expression of suprabasin-derived peptides in major human organs. Human tissue array sections were immunohistochemically stained with anti-SBSN_HUMAN[225–237] (**a**–**n**), anti-SBSN_HUMAN[243–259] (**a**′–**n**′) or anti-SBSN_HUMAN[279–295] (**a**″–**n**″) antibodies at 1:1000 dilution. (**a**–**a**″) cerebral cortex, (**b**–**b**″) pituitary gland, (**c**–**c**″) thyroid, (**d**–**d**″) stomach, (**e**–**e**″) liver, (**f**–**f**″) small intestine, (**g**–**g**″) colon, (**h**–**h**″) lung, (**i**–**i**″) heart, (**j**–**j**″) kidney, (**k**–**k**″) pancreas, (**l**–**l**″) adrenal gland, (**m**–**m**″) striated muscle, and (**n**–**n**″) skin (magnification, × 200 for all panels).

Specific polyclonal antibodies raised against SBSN_HUMAN[225–237], SBSN_HUMAN[243–259] and SBSN_HUMAN[279–295] were used for immunohistochemical analysis of human tissues. Immunoreactive SBSN_HUMAN[225–237] and SBSN_HUMAN[279–295] were concomitantly present throughout major human organs, including the central nervous system, stomach, liver, lung, heart, kidney and adrenal gland (Fig. 8a–n and a″–n″). The expression of SBSN_HUMAN[243–259] was not ubiquitous but was abundantly detected in the liver and pancreas (Fig. 8a′–n′). The widespread colocalization of SBSN_HUMAN[225–237] and SBSN_HUMAN[279–295] and the distinct distribution of SBSN_HUMAN[243–259] indicated that translated prosuprabasin transcripts may be processed into respective fragments and present in various human tissues. We next searched for cultured human cells that express SBSN_HUMAN[225–237], SBSN_HUMAN[243–259] and SBSN_HUMAN[279–295]. Confocal immunofluorescence microscopy revealed the expression of SBSN_HUMAN[225–237] and SBSN_HUMAN[243–259] in HAoSMCs, a human monocyte cell line (THP1), human macrophage cells, a human keratinocyte cell line (HaCaT) and a human hepatocellular carcinoma cell line (HepG2) (Supplementary Fig. S1). These results suggest a potential modulatory role for SBSN_HUMAN[225–237] and SBSN_HUMAN[243–259] at sites of inflammation, such as in the vasculature.

## Discussion

In this report, we describe a peptidomic strategy for non-tryptic plasma, which successfully identified a large number of distinct native peptides in human peripheral circulation. Using this peptidomic resource, we performed functional validation studies of synthesized peptides and ultimately unraveled the potent biological activities of three endogenous peptides. We did not employ proteomic differential display analysis or search for factors that showed distinct plasma level differences between healthy and disease samples; this is because most endogenous bioactive peptides are secreted locally and exert their effects in endocrine and/or paracrine fashions without significantly altering plasma concentrations or tissue expression levels. Therefore, we created the current strategy to first accurately identify as many native peptide sequences as possible that are universally present in healthy human peripheral circulation, and to functionally screen many of these factors to discover the most powerful endogenous regulators. We tested the ability of the initial series of 95 peptides to elicit intracellular responses in a cell culture model system and to modulate spontaneous animal behaviors. We subsequently performed extensive validation studies to confirm their functions and their systemic expressions in human tissues and cells. These initial attempts resulted in the discovery of three novel bioactive peptides derived from the same precursor protein, suprabasin, which have potent and desired biological activities.

Our results revealed SBSN_HUMAN[225–237] and SBSN_HUMAN[243–259] to be novel endogenous stimulators of NF-κB and inducers of cytokines, chemokines and growth factors, which are well-known to play pivotal roles in inflammation and cancer biology. NF-κB is an inducible dimeric transcription factor^27^ and upon dissociation from the inhibitor protein, IκB-α, which is degraded by the 26S proteasome, it is translocated to the nucleus and binds to specific DNA sequences in the promotor regions of multiple genes^28^. The current study further showed that SBSN_HUMAN[225–237] and SBSN_HUMAN[243–259] induced the expressions of immediate early oncogenes, Egr1 and c-Myc, stimulated cell proliferation and suppressed apoptosis of vascular smooth muscle cells. Egr1 and c-Myc are rapidly upregulated in response to growth promoting factors to transduce the proliferating signals^29–31^ and play key roles in mediating apoptosis in many cell types^32–35^. We found previously that bioactive peptides, which induce c-Myc oncogene and mitogenesis, suppress serum starvation-induced apoptosis in several cell types including vascular smooth muscle cells^36–39^. Our results revealed that SBSN_HUMAN[225–237] and SBSN_HUMAN[243–259] have similar functions, supporting their roles as mitogenic and anti-apoptotic factors for vascular smooth muscle cells. Such activities of SBSN_HUMAN[225–237] and SBSN_HUMAN[243–259] suggest their potential contribution to the previously described functions of the suprabasin gene on cancer progression, invasion and metastasis^15–20^. Potential involvement of these suprabasin-derived peptides in the pathophysiology of human diseases is further indicated by the following results. First, LC–MS/MS analysis utilizing XICs generated by plasma peptides and synthetic stable-isotope-tagged peptides revealed significant nM concentrations in human plasma. Second, immunohistochemistry using specific antibodies detected their presence in a variety of human tissues and cultured cells, including human macrophages. Third, SBSN_HUMAN[225–237] and SBSN_HUMAN[243–259] bound to the surface of cells and induced intracellular responses in human and rat vascular smooth muscle cells. These results provide sufficient rationale for investigating the involvement of SBSN_HUMAN[225–237] and SBSN_HUMAN[243–259] in the vasculature and at sites of inflammation and tumor progression because NF-κB and immediate early oncogenes are profoundly involved in the pathophysiology of a variety of human diseases.

Intraperitoneal administration of very low doses of SBSN_HUMAN[279–295] suppressed food and water intake and induced spontaneous locomotor activities in ad libitum watered and fed mice. The effective doses at which SBSN_HUMAN[279–295] induced significant anorexigenic and antidipsogenic effects were far lower than those of previously reported bioactive peptides that exert similar suppression of food intake. Glucagon-like peptide 1^40,41^, leptin^42^ and cholecystokinin^43^, for example, reduce food intake in mice after intraperitoneal administration of 100 µg/kg, 25 mg/kg and 4 µg/kg, respectively, which correspond to approximately 6 to 8 orders of magnitude higher doses than those we observed with SBSN_HUMAN[279–295]. In the current study, we carefully measured food and water consumption and locomotor activities using a system that accurately measures these spontaneous behaviors over the entire dark phase of the diurnal cycle^24,25^ because the majority of spontaneous rodent activities occur during the dark phase^44^. Considering the plasma concentration of SBSN_HUMAN[279–295] determined to be 1.5 nM using a stable isotope-tagged peptide technique and that very low doses exerted anorexigenic and anti-dipsogenic effects, endogenous SBSN_HUMAN[279–295] is potentially involved in the maintenance and/or regulation of eating and drinking behaviors.

The efficiency and accuracy of our current success in enriching and identifying low-molecular-weight native peptides embedded in a vast amount of plasma proteins is analogous to correctly selecting and identifying several hundred sesame seeds randomly scattered in an Olympic-size swimming pool filled with an enormous variety of beans. Our technique to enrich native peptides in plasma is comparable to removing the ‘beans’ from the pool to as few as 100,000 without significantly losing or damaging the ‘sesame seed’ population, while subsequent fractionation of the sesame-seed-rich fluid enables accurate identification of these sesame seeds. Thus, our unique non-tryptic peptidomic strategy using a single drop of human plasma enables us to commence high throughput functional screening using the identified endogenous “orphan ligands” library.

In conclusion, our current approach identified a huge number of endogenous peptide sequences far more rapidly than previous efforts and our initial trial to screen the biological activities of 95 synthetic peptides resulted in the discovery of three novel peptide hormones. Our human plasma peptidome strategy facilitated the discovery of novel bioactive peptides and biomarkers far more efficiently than other approaches; therefore, we propose to use either UniProt entry names or protein names with their amino acid positions, such as SBSN_HUMAN[279–295] or human suprabasin(279–295), rather than designating new names to each new bioactive peptide discovered.

## Methods

### Peptide extraction and reductive alkylation of human plasma

Blood samples were collected from four healthy volunteers into vacutainers containing Na_2_-EDTA and immediately separated in a refrigerated centrifuge at 1000*g* for 20 min. Aliquots were immediately flash-frozen in liquid nitrogen and stored at − 80 °C until processing. Thawed plasma was processed according to the differential solubilization method, as described previously^11,12^, but with the following modifications. A 50-μL plasma sample was diluted 1:2 with 100 μL denaturing solution (7 M urea, 2 M thiourea and 20 mM dithiothreitol), slowly dropped into 2 mL ice-cold acetone, with stirring at 4 °C for 1 h and then centrifuged at 19,000*g* for 15 min at 4 °C. The precipitate was resuspended in 1 mL 80% acetonitrile containing 12 mM HCl, mixed at 4 °C for 2 h and centrifuged again at 19,000*g* for 15 min at 4 °C. The low molecular weight peptides fraction in the supernatant was lyophilized and stored at − 80 °C until use. Efficient depletion of plasma high abundant proteins was confirmed using tricine SDS–polyacrylamide gel electrophoresis of the eluted samples as described^11,12^. Lyophilized peptides were re-dissolved in a solution of 1 × Invitrosol (Life Technologies, CA, USA) and 100 mM ammonium hydrogen carbonate^22^. Cystine disulfides were reduced by incubation with 2 μL 200 mM dithiothreitol for 1 h at 37 °C and alkylated with 2 μL 600 mM iodoacetamide for 30 min at room temperature.

### Prefractionation of peptides by RP-HPLC

Re-dissolved peptides with or without reductive alkylation treatment, corresponding to either 200 or 10–20 μL of original plasma, respectively, were injected onto a 2.0-i.d. × 100-mm C18 RP column (Cadenza CD-C18; Imtakt Corp., Kyoto, Japan) attached to an HPLC system (Nanospace SI-2; Shiseido Fine Chemicals, Tokyo, Japan). The flow rate of the mobile phase was set at 200 μL/min and the solvent composition was programmed to change during a 95-min cycle with varying ratios of mobile phase (A) 0.1% trifluoroacetic acid (TFA) and (B) 0.08% TFA/90% acetonitrile: 5% B (0–10 min), 5–45% B (10–63 min), 45% B (63–74 min), 45–95% B (74–74.1 min), 95% B (74.1–79.9 min), 95–5% B (79.9–80 min) and 5% B (80–95 min). The elution was monitored at 218 nm with a photodiode array detector (Shiseido Fine Chemicals). Extracted peptides were separated by RP-HPLC, collected at 1 min intervals, and combined into either 8 or 12 fractions by the cyclic sample pooling method^21^. To combine separated eluates into eight fractions, every 8th eluate collected from 11 to 67 min (56 fractions) was combined; i.e., seven fractions, Nos. 1, 9, 17, 25, 33, 41 and 49, were combined to make the cyclic sampled fraction No. 1. To combine eluted samples into 12 fractions, every 12th eluate collected from 11 to 71 min (60 fractions) was combined, such that fractions No. 1, 13, 25, 37 and 49 were combined. Eluates from fractions collected from 72 to 76 min were also combined to be analyzed as the 13th fraction. All HPLC fractions were then lyophilized and re-dissolved in 20 μL 500 ng/mL NV10 (AMR, Tokyo, Japan) and 8 μL of this peptide solution, which originally derived from 200 μL plasma, was analyzed by LC–MS/MS.

### Desalting using STAGE tips

Samples without RP-HPLC prefractionation were desalted using stop and go extraction tips (STAGE tips)^45^ filled with Empore™ C18 sealant (3 M, MN, USA). The peptides were eluted with 40 μL 80% acetonitrile containing 0.1% TFA. The sample solutions were then lyophilized and re-dissolved in 20 μL 500 ng/mL NV10 for LC–MS/MS analysis.

### LC–MS/MS analysis

RP-HPLC prefractionated and non-fractionated samples were injected onto a C18 0.075 × 20 mm trap column (Acclaim PepMap 100; Thermo Fisher Scientific, San Jose, CA, USA) and then eluted onto a C18 0.075 × 120 mm analytical column (Nano HPLC Capillary Column; Nikkyo Technos, Tokyo, Japan) configured to an EASY-nLC 1000 HPLC system (Thermo Fisher Scientific). The flow rate of the mobile phase was 300 nL/min; mobile phase (A) consisted of 0.1% formic acid and mobile phase (B) consisted of 0.1% formic acid/90% acetonitrile. After sample introduction, the ratio of these solvents was programmed to change in 54-, 70-, 90-, 120-, 165- and 215-min cycles. Separated peptides were subjected to Q-Exactive™ mass spectrometry (Thermo Fisher Scientific) operated in data-dependent mode to automatically switch between full-scan MS and MS/MS acquisition. The 12 most intense full-scan peaks were selected with an isolation window of 1.6 or 2.4 Da. Typical mass spectrometric conditions were as follows: spray voltage, 2 kV; no sheath or auxiliary gas flow; heated capillary temperature, 250 °C.

### Database searching

Raw LC–MS/MS data obtained from all 189 runs were classified to four MS groups. All LC–MS/MS acquisition data in each of MS group was searched together against the SwissPro_2020_03.fasta database (selected for Homo sapiens; 20,365 entries) using PEAKS database search algorithm (Bioinformatics Solutions, Waterloo, Canada). The PEAKS Studio (version X) performed peak picking, de-isotoping, charge deconvolution of fragment ions and a de novo peptide sequencing-based database search from MS and MS/MS spectra of peptides. The search parameters were as follows: enzyme, no enzyme; fixed modification, carbamidomethyl (C, only for samples with reductive alkylation); variable modifications, acetyl (N-term, K), amidated (C-term), pyro-glu from Q (Q), oxidation (M), carbamidomethyl (N-terminal, only for samples with reductive alkylation); peptide ion mass tolerance, 6 ppm; fragment ion mass tolerance, 0.02 Da. The PTM algorithm of PEAKS Studio was applied to identify variable modifications and substitutions^46–48^. The FDR was set as 1%.

### Peptide synthesis and dissolution

To explore the biological functions of the identified sequences, we screened the bioactivities of selected peptides having the following characteristics for chemical synthesis: (1) uniquely assigned to precursor proteins, (2) having a gene name, (3) encoded by secretory proteins as defined by SwissProt keywords, (4) between 5 and 39 amino acid residues, (5) without any substitutions or modifications. For the initial round of chemical synthesis, 105 sequences were synthesized (SCRUM. Tokyo, Japan) and reconstituted to 2–10 × 10^–3^ M in 10% acetonitrile/0.1%TFA. After dilution to 1:1000, the synthesized peptides were analyzed by LC–MS/MS to confirm their high purity and solubility.

### Measurements of plasma peptide levels using stable isotope-labeled peptides

The following three stable isotope-labeled peptides were synthesized by Scrum (Tokyo, Japan) using l-lysine-*N*-9-fluorenylmethoxycarbonyl (^13^C_6_, 98%; ^15^N_2_, 98%) and l-arginine-*N*-9-fluorenylmethoxycarbonyl (^13^C_6_, 98%; ^15^N_4_, 98%): SI-SBSN_HUMAN[225–237], GQGIHHAAGQVGK, SI-SBSN_HUMAN[243–259], GQGAHHAAGQAGNEAGR, and SI-SBSN_HUMAN[279–295], GQGVHHTAGQVGKEAEK. The underlined amino acids contained the stable isotope. The serially diluted stable isotope-labeled peptides, SI-SBSN_HUMAN[225–237], SI-SBSN_HUMAN[243–259] and SI-SBSN_HUMAN[279–295], were spiked into plasma samples from healthy volunteers and, after extraction using improved differential solubilization method and prefractionation, LC–MS analysis was performed to generate the extracted ion chromatogram (XIC) intensities for the respective endogenous peptides, SBSN_HUMAN[225–237], SBSN_HUMAN[243–259] and SBSN_HUMAN[279–295], and the corresponding stable-isotope-labeled peptides, SI-SBSN_HUMAN[225–237], SI-SBSN_HUMAN[243–259] and SI-SBSN_HUMAN[279–295] (Fig. 2). The plasma concentrations were then extrapolated from the XICs generated using the respective endogenous peptides and the corresponding spiked stable isotope-labeled peptides as described^26^.

### Cell culture

Human aortic smooth muscle cells (HAoSMCs) from Promocell (Heidelberg, Germany), rat aortic smooth muscle cells (A-10, CRL 1476) from American Type Culture Collection (USA), human monocytic leukemia cells (THP1) and human hepatocellular carcinoma cells (HepG2) from Riken CELL BANK (Ibaraki, Japan), and human keratinocyte cells (HaCaT) from Cell Lines Service (Eppelheim, Germany) were purchased. Cells were cultured using the appropriate medium and supplements recommended by the suppliers. Successive experiments using HAoSMCs were performed with passage 4–8 cultures. Human monocytes were separated from peripheral blood samples using Lymphocyte Separation Solution (Nacalai Tesque, Kyoto, Japan) as described^49^ and incubated in RPMI1640 medium containing 10% fetal bovine serum for 3–4 days for differentiation into macrophages.

### Determination of intracellular free Ca^2+^ concentration [Ca^2+^]_i_

Confluent cultures of A10 cells and HAoSMCs in non-coated 96 well black, clear bottomed plates were deprived of serum for 16 h and then incubated in Hank’s Balanced Salt Solution (HBSS) with Fluo-4 acetoxymethyl ester (Fluo-4 AM) (Dojindo Molecular Technologies, Kumamoto, Japan) at 37 ℃ for 30 min. Fluo-4 AM-loaded cells were washed three times with HBSS, then suprabasin-derived peptides were applied. Cell fluorescence was read at the indicated times with an excitation wavelength of 485 nm and an emission wavelength of 535 nm using a POWERSCAN HT microplate reader (BioTek Instruments, Winooski, VT, USA)^8,50^.

### Evaluation of cell proliferation and apoptosis

A10 cells seeded into 12-well cluster dishes (2.0 × 10^5^ cells/well) were deprived of serum for 24 h and further incubated in the presence or absence of SBSN_HUMAN[225–237] or SBSN_HUMAN[243–259] for the indicated times. Cells were then trypsinized, and cell numbers measured using an EVE automatic cell counter (NanoEnTek Inc, Seoul, Korea)^50^.

HAoSMCs were cultured to subconfluency and serum deprived for 9 h in the presence or absence of SBSN_HUMAN[225–237] (10^–6^ M) or SBSN_HUMAN[243–259] (10^–6^ M). The terminal deoxynucleotidyl transferase mediated dUTP nick end labeling (TUNEL) assay was performed using an ApopTag Fluorescein Direct In Situ Apoptosis Detection Kit (EMD Millipore, USA)^51^. DNA fragmentation was detected by laser scanning confocal microscopy using an LSM710 confocal microscope (Carl Zeiss, Jena, Germany).

### Real-time RT-PCR

Total mRNA was isolated from HAoSMCs using TRIzol reagent (Invitrogen, Carlsbad, CA, USA), reverse transcribed with a first-strand cDNA synthesis kit (Takara Bio, Shiga, Japan), and quantified using a CFX96 Touch™ Real-Time PCR Detection System (Bio-Rad Laboratories)-based RT-PCR protocol using KAPA SYBER (Nippon Genetics, Tokyo, Japan) as described^52^. After reverse transcription, the reaction mixtures were denatured at 94 ℃ for 3 min followed by 40 cycles of PCR at 94 ℃ for 10 s, 55 ℃ for 10 s, 72 ℃ for 30 s. PCR primers were synthesized by Eurofins Genomics (Tokyo, Japan) and their sequences are shown in Supplementary Table S3.

### Peptide binding to the surface of cells

A10 cells and HAoSMCs plated on glass coverslips were deprived of serum for 16 h and incubated for 30 min with each FAM-labeled suprabasin-derived peptide (10^–6^ M). Cells were then washed twice with PBS and fixed with 4% paraformaldehyde for 30 min. The nuclei were counterstained using DAPI Fluoromount-G (SouthernBiotech, Birmingham, AL, USA) and fluorescence was detected using an LSM710 confocal microscope (Carl Zeiss) as described^53,54^. For quantification of SBSN_HUMAN[225–237]- and SBSN_HUMAN[243–259]-binding to cultured cells, confluent cultures of A10 cells and HAoSMCs in non-coated 96 well black plates were incubated with serum-starved medium for 16 h, and further incubated for 5, 15, 30, 45, 60, 75, 90, or 105 min after addition of 10^–6^ M FAM-labeled SBSN_HUMAN[225–237] or SBSN_HUMAN[243–259]. Concentration response relationships for FAM-SBSN_HUMAN[225–237] and FAM-SBSN_HUMAN[243–259] were examined by incubating serum-starved A10 cells and HAoSMCs for 60 min in the presence and absence of various doses of FAM-SBSN_HUMAN[225–237] or FAM-SBSN_HUMAN[243–259]. Cells were washed three times with HBSS and fluorescence was measured using a POWERSCAN HT plate reader (BioTek Instruments). Cell-bound-specific fluorescence was calculated by subtracting non-specific adherent fluorescence arising from peptide material bound to the inside wall of the 96 well plate from the total fluorescence detected in each well^53^.

### Cytokine array

Confluent HAoSMCs were deprived of serum for 16 h, incubated with either vehicle, SBSN_HUMAN[225–237] (10^–7^ M) or SBSN_HUMAN[243–259] (10^–7^ M) for 24 h. Medium was clarified by centrifugation at 230*g* for 3 min at 4 ℃, and immediately applied to Proteome Profiler Human XL Cytokine Arrays according to the manufacturer’s instructions (R&D Systems, MN, USA). Cytokine array signals were detected using an ImageQuant LAS 4000 digital imaging system (GE Healthcare, Tokyo, Japan)^54^ and quantified using ImageJ software (http://rsb.info.nih.gov/ij/)^55^. Values from duplicate spots were averaged, and the relative signals calculated in SBSN_HUMAN[225–237]- and SBSN_HUMAN[243–259]-treated samples were compared with vehicle treated cells.

### LC–MS/MS identification of bioactive proteins released from cultured cells

Five hundred microliters of culture supernatant of serum-starved HAoSMCs were lyophilized and dissolved in 20 µL 200 mM triethylammonium bicarbonate/12 mM sodium deoxycholate/12 mM sodium lauryl sulfate, mixed for 10 min at room temperature and sonicated for 10 min at 4 °C. Samples were centrifuged at 19,000*g* for 15 min at 4 °C, and 20 µL of the supernatant was digested essentially as described^56^ with the following modifications. Two microliters of 200 mM Tris (2-carboxylethyl) phosphine hydrochloride and 120 mM triethylammonium bicarbonate were added to samples and incubated at 50 °C for 30 min. After adding 2 µL of 375 mM iodoacetamide, the samples were incubated in the dark for 30 min. Two microliters of Lys-C (100 ng/µL) and 2 µL of trypsin (100 ng/µL) were added to each sample, which were then incubated at 37 °C for 18 h. Thirty microliters of 10% acetonitrile and 15 µL of 5% TFA were added to each digest, centrifuged at 19,000*g* for 15 min and the supernatants recovered.

Samples were desalted using stop and go extraction tips (STAGE tips) filled with Empore™ C18 sealant (Octadecyl) (3 M, Saint Paul, MN, USA). Peptides were eluted with 80 μL of 70% acetonitrile containing 0.1% TFA. The sample solution was then lyophilized and re-dissolved in 20 μL 3% acetonitrile/0.1% formic acid for LC–MS/MS analysis.

Samples were injected onto a C_18_ 0.075 × 120 mm analytical column (Nano HPLC Capillary Column; Nikkyo Technos, Tokyo, Japan) attached to an EASY-nLC 1000 HPLC system (Thermo Fisher Scientific). The mobile phase consisted of 0.1% formic acid and 90% acetonitrile (solvent A), and the mobile phase gradient was programmed as follows: 0–5% A (0–2 min), 5–25% A (2–42 min), 25–55% A (42–56 min), 55–95% A (56–57 min), and 95% A (57–60 min). Separated peptides were subjected to analysis on a Q-Exactive™ instrument (Thermo Fisher Scientific) operated in data-dependent mode to automatically switch between full-scan MS and MS/MS acquisition. Full-scan mass spectra (*m/z* 350–900) were acquired on an Orbitrap instrument (Thermo Fisher Scientific) with 70,000 resolution at *m/z* 200 after accumulation of ions to a 3 × 10^6^ target value. The 20 most intense peaks with charge states more than two were selected from the full scan with an isolation window of 2.4 Da and fragmented in the higher energy collisional dissociation cell with a normalized collision energy of 27%. Tandem mass spectra were acquired on the Orbitrap mass analyzer with a mass resolution of 35,000 at *m/z* 200 after accumulation of ions to a 5 × 10^5^ target value. The ion selection threshold was 2 × 10^3^ counts, and the maximum allowed ion accumulation times were 100 ms for full MS scans and 50 ms for tandem mass spectra. Typical mass spectrometric conditions were as follows: spray voltage, 2 kV; no sheath and auxiliary gas flow; heated capillary temperature, 250 °C; and dynamic exclusion time, 30 s.

Database searches were performed using the SEQUEST algorithm^57^ incorporated in Proteome Discoverer 1.4.0.288 software (Thermo Fisher Scientific). The search parameters were as follows: enzyme, trypsin; variable modification, oxidation (M); static modification, carbamidomethyl (C); peptide ion mass tolerance, 6.0 ppm; fragment ion mass tolerance, 0.02 Da. The false discovery rate for peptide identification was set at 1%.

### In vivo analysis of water/food intake and spontaneous locomotor activity

Adult male C57BL/6J mice weighing 25–30 g (CLEA Japan, Tokyo, Japan) were maintained essentially as described^25,58^, in a controlled temperature environment (22–25 °C), with a 12-h light–dark cycle (lights on 08:00–20:00), and with free access to food (standard laboratory chow; CE2, CLEA Japan, Tokyo, Japan) and water. After at least 7 days of habituation with saline injections on weekdays, mice were intraperitoneally injected with indicated doses of synthetic peptides dissolved in 100 μL ddH_2_O or with 100 μL ddH_2_O alone using a 27 G needle without anesthesia approximately 30 min before the start of the dark period. Water and food intake and the locomotor activity were recorded using a simultaneous monitoring system (ACTIMO-100 M combined with MFD-100, Shinfactory, Fukuoka, Japan) essentially as described^24,25^. In brief, the monitoring system used beam sensors located every 2 cm along the floor of the enclosure and ACTIMO-DATA software (Shinfactory, Fukuoka, Japan) to detect animal movements. Data were imported in real-time using the Spike2 analysis program (Cambridge Electronic Design, Cambridge, UK). Water intake was measured by a drop counting system. A food container was designed to prevent mice from dragging food into their bedding and to avoid spillover and the minimum quantity of measurable food using the microbalance was 0.01 g. Water and food intake were recorded simultaneously every 3 min. Mice were housed in individual chambers for 3 days for them to become familiar with the recording environment. The experiments were performed during the dark phase (20:00–08:00) in a room that was completely isolated from external noises.

### Production and purification of peptide antibodies

Specific antibodies against SBSN_HUMAN[225–237], SBSN_HUMAN[243–259] and SBSN_HUMAN[279–295] were raised and purified essentially as described previously^10^, but with the following modifications. Two micrograms each of [Cys^0^]-PEG-GQGIHHAAGQVGK, [Cys^0^]-PEG-GQGAHHAAGQAGNEAGR, or [Cys^0^]-PEG-GQGVHHTAGQVGKEAEK were chemically synthesized. Approximately half of each was pretreated with a protein crosslinking and fixation reagent and then mixed with the remaining respective 1 μg of untreated peptide. Each peptide was then coupled to maleimide-activated mariculture keyhole limpet hemocyanin (Pierce) and used to immunize Japanese white rabbits on days 1, 15, 29, 43 and 57. Blood was collected prior to the first injection and on days 36, 50 and 64 post-injection and antibody titer was determined by ELISA. The polyclonal antisera were purified using a Melon™ Gel IgG Spin Purification Kit (Thermo Fisher Scientific) to remove non-relevant proteins that are often present in high abundance^10^.

### Immunoblotting

Western blotting was performed essentially as described^53,59^. Confluent HAoSMCs (1.2 × 10^6^ cells) deprived of serum for 16 h were incubated with SBSN_HUMAN[225–237] or SBSN_HUMAN[243–259] for the indicated time, washed three times with phosphate-buffered saline (PBS) and solubilized in RIPA Lysis and Extraction Buffer (Thermo Fisher Scientific) containing 10 μM protein inhibitors (Thermo Fisher Scientific). Extracted protein samples were loaded onto 4–20% gradient polyacrylamide gel (Bio-Rad Laboratories) and transferred to PVDF membranes (Immune-Blot or Trans-Blot Turbo™ Mini PVDF Transfer Packs, Bio-Rad). After blocking with Blocking One (Nacalai Tesque), membranes were incubated with an anti-IκB-α antibody (1:3000, Abcam, Cambridge, UK) or an anti-β-actin antibody (1:3,000, Abcam) overnight at 4 ℃, extensively washed, and incubated with a peroxidase-coupled secondary antibody (1:10,000, BioRad) for 1 h at room temperature. Protein bands were detected using an enhanced chemiluminescence system (ECL prime, GE Healthcare). The signals of each blot were visualized and quantitatively analyzed using ImageQuant LAS 4000 (GE Healthcare)^54^.

### Immunofluorescence staining

For assessing NF-κB translocation, subconfluent HAoSMCs, deprived of serum for 16 h, were incubated with SBSN_HUMAN[225–237] (10^–7^ M) or SBSN_HUMAN[243–259] (10^–7^ M) for 2 h. The cells were then fixed with 4% paraformaldehyde for 15 min, blocked with Blocking One (Nacalai Tesque), incubated overnight with an anti-p65 antibody (1:500, Santa Cruz biotechnology, CA, USA) at 4 ℃, and visualized by a 30 min incubation with a goat anti-rabbit Alexa Fluor 488 secondary antibody (1:2000, Abcam).

For the detection of cytokine expression, HAoSMCs, deprived of serum and incubated with SBSN_HUMAN[225–237] (10^–7^ M) or SBSN_HUMAN[243–259] (10^–7^ M) for 24 h were stained with either an anti-IL6 (1:500, Proteintech), anti-HGF (1:500, Abcam), or anti-VEGF (1:500, Abcam) antibody and visualized by a 30 min incubation with a goat anti-rabbit Alexa Fluor 594 secondary antibody (1:1000, Abcam) at 37 ℃.

The expression of the three suprabasin-derived peptides was also analyzed in human-derived cells in culture. Cells were washed twice with PBS, fixed with 4% paraformaldehyde for 15 min, blocked with Blocking One (Nacalai Tesque), incubated at 37 ℃ for 30 min with specific antibodies against SBSN_HUMAN[225–237], SBSN_HUMAN[243–259] or SBSN_HUMAN[279–295], diluted to 1:500 with 1% BSA and 0.1% sodium azide in PBS, and visualized by a 30 min incubation with a goat anti-rabbit Alexa Fluor 594 secondary antibody (1:1000, Abcam) at 37 ℃. Cell nuclei were counterstained using DAPI Fluoromount-G (SouthernBiotech). Laser scanning confocal microscopy was performed using an LSM710 confocal microscope (Carl Zeiss)^53,54^.

### Immunohistochemistry

An antibody cross-reactivity tissue array, MNO341 (US Biomax, Rockville, MD, USA), which contained 33 tissue types mostly from surgical resection, was used to examine the tissue distribution of each peptide. The tissue preparations were deparaffinized, rehydrated, and autoclaved in pH 6.0 citric acid buffer for antigen retrieval. The preparations were blocked using an endogenous Avidin Biotin Blocking Kit (Nichirei Bioscience, Tokyo, Japan) and then incubated overnight at 4 ℃ with either anti-SBSN_HUMAN[225–237] IgG, anti-SBSN_HUMAN[243–259] IgG, anti-SBSN_HUMAN[279–295] IgG or control non-immune IgG purified from preimmune serum (1:1000 dilution). After washing in PBS, the samples were incubated with a biotinylated anti-rabbit IgG antibody (1:3000, Abcam) for 60 min at room temperature. Antibody binding was visualized by the avidin–biotin-complex peroxidase method using the Vectastain ABC kit (Vector Laboratories, Burlingame, CA, USA) with diaminobenzidine tetrahydrochloride (Nacalai Tesque). The tissue preparations were stained with hematoxylin (Muto Pure Chemicals CO, Tokyo, Japan)^60^.

### Statistical analysis

Data are presented as the mean ± S.E.M and were analyzed using GraphPad Prism software 5.02. Differences between groups for gene expression and cell number were compared using the Kruskal–Wallis test, followed by Dunn’s post hoc test. Comparisons between two groups were performed using Mann–Whitney U tests. *P* < 0.05 was considered statistically significant.

### Ethics approval and consent to participate

All animal experimental procedures were approved by the Animal Experimentation and Ethics Committee of Kitasato University School of Medicine (2016-144). Procedures were performed in accordance with the guidelines for animal experiments of Kitasato University School of Medicine. The study protocol using human blood samples was approved by the Ethics Committees of Kitasato University Hospital/School of Medicine (C19-245). All study methods were undertaken in accordance with the relevant guidelines and regulations of this organization as well as the Ethical Guidelines for Medical and Health Research Involving Human Subjects in Japan. Written informed consent was obtained from all healthy volunteers who provided blood samples.

## Supplementary Information

Supplementary Information.
