# Supplementary material for: Suprabasin-derived bioactive peptides identified by plasma peptidomics
Source: Sci Rep. 2021 Jan 13;11:1047. doi: 10.1038/s41598-020-79353-4 (PMC7806982; doi:10.1038/s41598-020-79353-4)

**Supplementary Information**

**Suprabasin-derived bioactive peptides identified by plasma peptidomics**

Tomomi Taguchi^1^, Yoshio Kodera^2,3^, Kazuhito Oba^1^, Tatsuya Saito^1,2^,

Yuzuru Nakagawa^2^, Yusuke Kawashima^2^, & Masayoshi Shichiri^1*^

^1^Department of Endocrinology, Diabetes and Metabolism, Kitasato University School of Medicine, 1-15-1 Kitasato, Minami-ku, Sagamihara, Kanagawa 252-0374, Japan

^2^Department of Physics, and ^3^Center for Disease Proteomics, Kitasato University School of Science, 1-15-1 Kitasato, Minami-ku, Sagamihara, Kanagawa 252-0373, Japan

Correspondence and requests for materials should be addressed to M.S. (shichiri@kitasato-u.ac.jp)


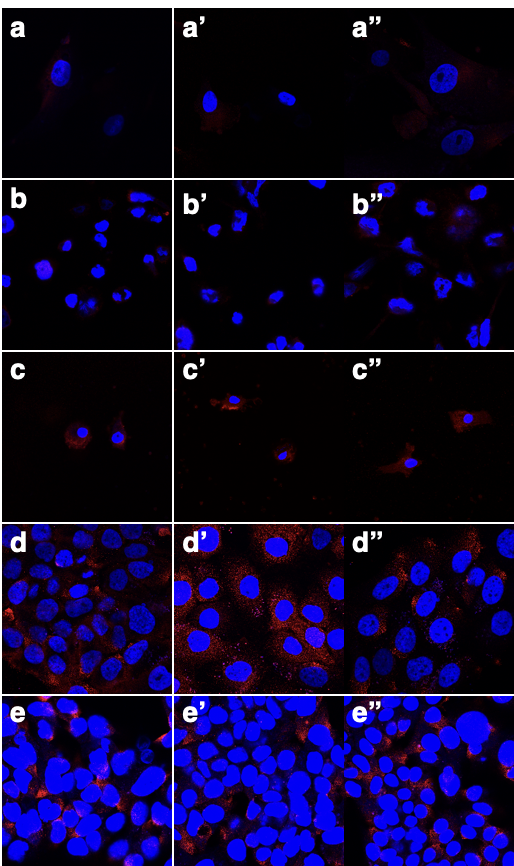


**Supplementary Figure S1. The expression of the suprabasin-derived peptides in cultured human cells**

Confocal fluorescence microscopy images of immunoreactive SBSN_HUMAN[225–237] (a–e), SBSN_HUMAN[243–259] (a’–e’) and SBSN_HUMAN[279–295] (a’–e’’) stained with their respective antibodies at 1:1000 dilution. (a–a’’) HAoSMCs, (b–b’’) THP1 cells, (c–c’’) human macrophages, (d–d’’) HaCaT cells, (e–e’’) HepG2 cells. The red signals corresponded to the expression of immunoreactive suprabasin-derived peptides. The nuclei were counterstained with DAPI (blue).

**Supplementary Table S1 List of synthetic peptides used for functional analyses**

**Supplementary Table S2 Proteins identified by LC-MS/MS analysis of serum-starved HAoSMC culture supernatant**

Protein Name Uniprot accession Gene name Uniprot MW×10^-3^ Coverage (%) Unique PSMs

entry name Peptide

Cystatin-C P01034 CST3 CYTC_HUMAN 15.8 30.8 4 5

Dickkopf-related protein 1 O94907 DKK1 DKK1_HUMAN 28.7 4.9 1 1

Dickkopf-related protein 3 Q9UBP4 DKK3 DKK3_HUMAN 38.4 22.9 6 7

Endoglin P17813 ENG EGLN_HUMAN 70.5 7.8 4 4

Hepatocyte growth factor P14210 HGF HGF_HUMAN 83.1 1.1 1 1

Interleukin-6 P05231 IL6 IL6_HUMAN 23.7 12.3 3 3

Macrophage colony-stimulating factor 1 P09603 CSF1 CSF1_HUMAN 60.1 5.4 3 3

Vascular cell adhesion protein 1 P19320 VCAM1 VCAM1_HUMAN 81.2 5.0 4 4

UniProt accession code, Gene Name, Uniprot entry name, and approximate molecular weight (MW) for each protein are listed together with sequence coverage rate (Coverage), peptide spectral matches (PSMs) and the number of unique peptides identified with a false discovery rate of 1% (Unique Peptide) in each protein sequence..

**Supplementary Table S3 Primer sequences for RT-PCR**

| **Primers** |  | **Sequence** |  |  |  |  |
| --- | --- | --- | --- | --- | --- | --- |
|  |  |  |  |  |  |  |
| β-Actin |  | Forward | CCAACCGCGAGAAGATGA | | |  |
|  |  | Reverse | CCAGAGGCGTACAGGGATAG | | |  |
| IL6 |  | Forward | GAAAGTGGCTATGCAGTTTGAA | | | |
|  |  | Reverse | GAGGTAAGCCTACACTTTCCAAGA | | | |
| DKK1 |  | Forward | ATGCGTCACGCTATGTGCT | | |  |
|  |  | Reverse | AAGACAAGGTGGTTCTTCTGGA | | | |
| ENG |  | Forward | ACCACTAGCCAGGTCTCGAA | | |  |
|  |  | Reverse | GATGCCTGGAGAGTCAGCTC | | |  |
| VEGF |  | Forward | CTGTCTAATGCCCTGGAGCC | | |  |
|  |  | Reverse | TTAACTCAAGCTGCCTCGCC | | |  |
| HGF |  | Forward | CCATGATACCACACGAACACAG | | | |
|  |  | Reverse | AGCGTACCTCTGGATTGCTT | | |  |
| PLAUR |  | Forward | TGCAACACCACCAAATGCAAC | | | |
|  |  | Reverse | TGCAGCTGTAACACTGGCG | | |  |
| CST3 |  | Forward | CACGTGTACCAAGACCCAGC | | |  |
|  |  | Reverse | TGCATAAGAGGTGATAGGCACAG | | | |
| OPN |  | Forward | TTTGCCTCCTAGGCATCACC | | |  |
|  |  | Reverse | GCTTCTGAGATGGGTCAGGG | | |  |
| c-MYC |  | Forward | GCTGCTTAGACGCTGGATTT | | |  |
|  |  | Reverse | TAACGTTGAGGGGCATCG | | |  |
| EGR1 |  | Forward | TCTGAACAACGAGAAGGTGCT | | | |
|  |  | Reverse | GGGCAGTCGAGTGGTTTG | | |  |
|  |  |  |  |  |  |  |

**Uncropped Figures**

Uncropped photographs of the protein blots shown in Figure 6g.

**Blot: IκB-α**

Left upper panel Right upper panel


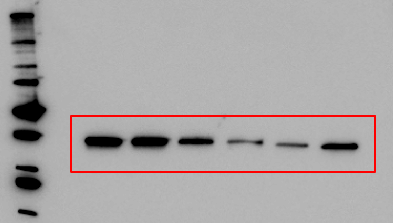

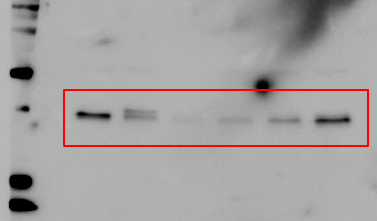


**Blot: β-Actin**

Left lower panel Right lower panel:


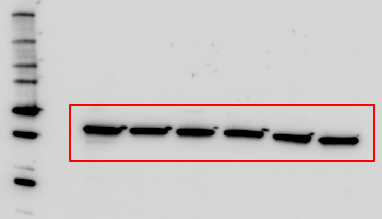

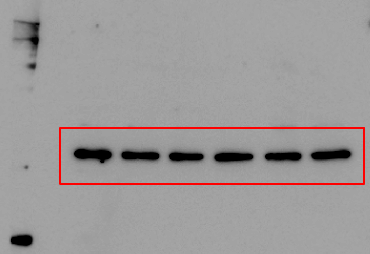

Supplement: Supplementary file 1 — Supplementary Information. [file 41598_2020_79353_MOESM1_ESM.docx]
